# Complementary and alternative therapy use in a regional radiation oncology treatment centre: Can staff knowledge, views, confidence and documentation be improved?

**DOI:** 10.1002/jmrs.344

**Published:** 2019-08-08

**Authors:** Lisa J. Delaney, Stephen J. Manley

**Affiliations:** ^1^ Department of Radiation Oncology North Coast Cancer Institute Lismore New South Wales Australia

**Keywords:** Alternative medicine, attitude of health personnel, complementary therapies, oncology, radiotherapy

## Abstract

**Introduction:**

Complementary and alternative therapies (CATs) are widely used by cancer patients but are infrequently disclosed and documented. This study aimed to improve radiation therapy staff knowledge, confidence, views and documentation of radiation oncology patients' use of CATs.

**Method:**

Participants completed a baseline questionnaire regarding their knowledge, confidence, views and documentation relating to patients' CAT use. An intervention was undertaken whereby participants attended an educational session and a CAT screening tool was implemented simultaneously. Participants immediately completed a post‐intervention questionnaire and later a 6‐month follow‐up questionnaire. A patient record audit was conducted to measure the documentation of CAT use pre‐ and post‐intervention.

**Results:**

From baseline to post‐intervention, there was a statistically significant shift in staff knowledge and confidence (*P *=* *0.001–0.01). The observed shift was sustained over a 6‐month period, (*P *=* *0.453–1.00). Participants' perceived views of CATs did not change as a result of the intervention (*P *=* *0.261–1.000). The post‐intervention audit compared to the baseline audit yielded a statistically significant increase in documentation. There was an increase in CAT use mentioned in patient records from 14% (15/108) to 40% (35/88) (*P *<* *0.001).

**Conclusions:**

The implementation of a screening tool and staff education increased radiation therapy staff knowledge of CATs and increased staff confidence when discussing CAT use with patients. Documentation of CATs in the patient record increased post‐intervention. These changes positively affected radiation therapy staff understanding the use of CATs by cancer patients.

## Background

Lismore is located in the Northern Rivers which is colloquially known as ‘The Rainbow Region' with many communities living an alternative lifestyle.^1^ Northern Rivers had the highest number of complementary and alternative therapy (CAT) practitioners of any rural NSW region, almost double the number of General Practitioners.^1^ Described as medicines, practices and products not considered part of conventional medicine, CATs can be ill‐defined.^2^, ^3^, ^4^, ^5^ Lismore's regional radiation oncology treatment centre provides cancer services for patients including radiation therapy. Clinical radiation oncology staff mentioned that patients ask about CATs, such as selenium. Staff said they felt their knowledge about CATs was low and they did not know where to find evidence‐based information. There were neither standardised departmental guidelines assisting with CAT discussions with patients, nor evidence‐based information provided.

A literature review was undertaken looking at cancer patients' CAT use with a specific focus on radiation therapy patients. Publications have shown up to 87% of Australian cancer patients are using CATs.^4^, ^6^, ^7^, ^8^, ^9^, ^10^ In terms of location, a higher incidence of CAT consumption and CAT practitioner use in Australian rural communities compared to their metropolitan counterparts has been reported.^1^, ^11^ Two studies explored CAT use in Australian regional radiation therapy centres showing the usage rates of 83% and 38%. These two studies also assessed the types of CATs used by patients, with Edwards et al reporting that vitamins, minerals, oils and herbs (67.8%) were the most commonly used category of CAT measured. Gillett et al reported that, of their nine CAT categories measured, vitamins (53%) and antioxidants (29%) were the most commonly used category of CATs by patients and herbs (18%) being fourth most commonly used.^2^, ^12^, ^13^

These studies had the most relevance, but with only a few radiation therapy specific publications identified, publications on CAT use in all cancer patients were incorporated. From the literature, it can be assumed that a high proportion of patients undergoing radiation therapy in Lismore are utilising CATs due to the centres rural locality servicing an alternative lifestyle population and the reported high use of CATs amongst both radiation oncology and cancer patients.^1^, ^2^, ^4^, ^6^, ^7^, ^8^, ^9^, ^10^, ^11^

### Disclosure

The reasons that cancer patients use CATs have been investigated, and include reducing the side effects of conventional treatment, to assist treatment, improve symptoms, increase quality of life, improve emotional and physical well‐being, to have a sense of control and less frequently to prevent recurrence, cancer control and boost immunity.^2^, ^7^, ^9^, ^13^, ^14^, ^15^ There are benefits from patients utilising CATs that can contribute to potential positive outcomes including the psychological benefit due to an improvement in outlook and optimism.^2^ Whilst most CATs, particularly psychosocial therapies, tend not to interfere with radiation therapy, some systemically administered or ingested therapies such as herbal or dietary therapies may have risks of adverse effects.^2^, ^6^, ^15^ For example, selenium is found naturally in food and is used by some patients to protect against cancer. When taken in high doses selenium supplements are toxic and upper daily intake limits are advised.^16^ Often CATs are utilised by patients without a physician's knowledge, subsequently CATs are not always discussed with the physician.^6^, ^7^, ^8^, ^12^ Open communication discussing CAT use is important as this affects the doctor–patient relationship and helps to make informed decisions regarding therapies.^8^, ^17^ Cancer patients frequently use CATs; however, there is a large disparity between CAT utilisation and disclosure rates.^4^, ^6^, ^7^, ^8^, ^9^, ^10^, ^12^ Gillet et al (2012) suggests only 40% of patients discussed CAT use with physicians whilst Pirri et al (2011), reported that 33–77% of patients did not disclose their CAT use.^9^, ^12^

### Education and knowledge

It has been acknowledged that there is a need to understand efficacy and interaction with conventional treatments and that there needs to be a greater awareness of the use of CATs in radiation therapy patients.^18^ Health professionals need to be informed and refer to current evidence, in order to assist patients with an informed judgement on CAT options.^7^, ^8^, ^12^, ^15^ There is a need to assist health professionals in providing this information through education.^8^ Lack of knowledge is one barrier and is exacerbated by a gap in the quality and rigour of evidence.^16^, ^19^

With greater knowledge of evidence comes increased opportunity to avoid risk of adverse reaction such as toxicity or potential interference with radiation therapy treatment.^2^, ^6^, ^15^, ^16^ If a professional is not aware of what CATs patients are taking, then potential adverse reactions cannot be discussed and therefore avoided.^6^, ^13^, ^15^ The use of CATs should not be ignored and due to the risks involved, health professionals are encouraged to ask patients about their CAT use.^20^

### Documentation

The importance of documenting CAT use is widely published and recommended.^4^, ^13^ If patients are not discussing CAT use, then clinicians are not able to address or document it. A recommendation from Gillet et al was CAT use ‘*should be specifically inquired about and recorded during the initial consult'*.^12^ A 2007 study of Australian radiation therapy centres found that only 44% obtain details of CAT use.^15^ A study by Edwards et al, introduced a self‐reported screening tool to identify CAT use in a regional setting and evaluate CAT use of their patients.^2^ Two publications reported from clinicians survey responses that they want to communicate with patients regarding CAT use and want greater access to evidence‐based information.^9^

The literature review revealed CATs are frequently utilised by cancer patients; however, disclosure and documentation is low and increasing staff evidence‐based knowledge is recommended.^6^, ^8^ Little is known about the confidence levels of staff within radiation therapy centres to discuss these therapies or how to educate staff and increase their knowledge. These identified gaps in the literature influenced the development of this study to provide a solution to assist our centre in addressing our patients' needs. The aim of this study was to improve radiation therapy staff knowledge, documentation, confidence and views of radiation oncology patients' use of CATs.

## Methods

A quantitative intervention study was conducted at Lismore's radiation oncology treatment centre during 2013–2015. All radiation oncology clinical staff disciplines were invited to participate, see Table 1. Participants were enrolled via email and were provided with a participant information sheet and a consent form to complete. Participation was voluntary and it was clearly stated no impact to their employment relationship would result and they could withdraw at any point.

**Table 1:** Participant group at each stage.

	Baseline	Post intervention	Follow‐Up
Allied health	4	4	3
Nurse	5	3	1
Radiation oncologist/Registrar	4	3	3
Radiation therapist	20	17	14
**Total**	**33**	**27**	**21**

**Figure 1:**
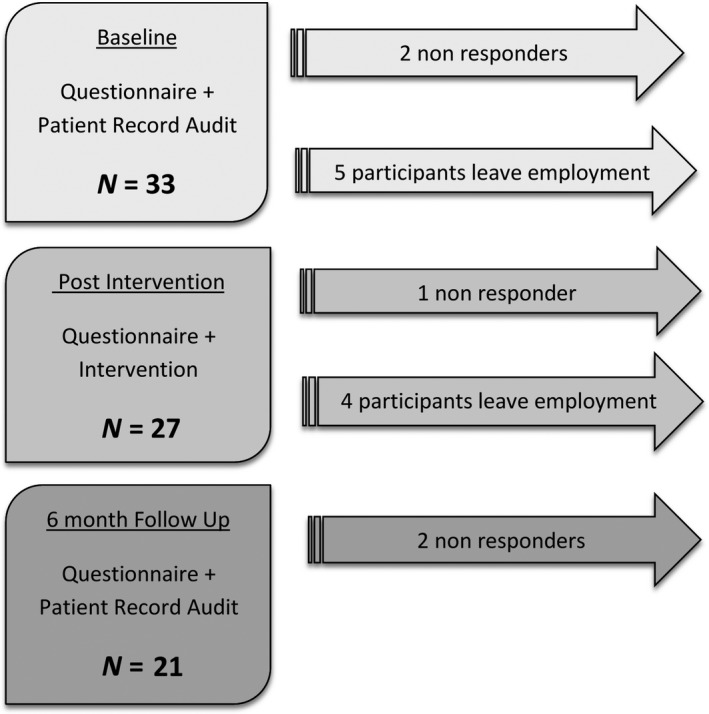
Project timeline and participant flow.

Baseline questionnaires were completed by participants and an audit of patient records for CAT documentation was conducted by the principal investigator. Participants attended the intervention education session and completed a post‐intervention questionnaire. Six months after the intervention a follow‐up questionnaire was completed by participants and the researcher conducted an audit of CAT documentation in patient records, see Figure 1.

### Sample profile

There were 33 participants at baseline from a potential pool of 35 clinical radiation oncology staff yielding a 94% (33/35) response rate. At post‐intervention and follow‐up, the response rate from eligible participants was 96% (27/28) and 91% (21/23) respectively, see Table 1 and Figure 1.

### Measurement instruments

#### Questionnaires

Questionnaires were developed by the principal investigator after an unsuccessful search for suitable pre‐existing validated instruments. The questionnaires were scrutinised by two experienced people in radiation therapy managerial roles and tested by an independent non‐participant radiation therapist from another centre. The questionnaires were the same across all three time points except for changes in tense for comprehension. Questions regarding the CAT screening tool that was introduced at the intervention were added in the post‐intervention and follow‐up questionnaires.

#### Screening tool

The CAT use screening tool was used with permission from a prior study by Edwards et al, which was based on a previously validated survey.^2^ A new item was added in the current electronic nursing new patient assessment to check if the screening tool was present.

#### Education session

The education session was created by the principal investigator using an extensive list of reputable evidence‐based resources. The one‐hour session was delivered several times to accommodate attendance and covered:

- A definition and overview of CATs
- Why CAT awareness is important in radiation therapy
- The national recommendations for CAT documentation and discussion
- How staff can discuss CAT use with patients effectively
- Evidence‐based literature summary of some CAT products, for example selenium and black cohosh
- A list of resources to aid staff to further advise and inform patients about CAT usage
- An overview of the CAT screening tool and information regarding its implementation

#### Audit

Patient records were audited pre‐ and post the education intervention. Mention of CATs in the patient notes by any staff member was counted. Head and neck patients were identified because they are routinely seen by the allied health team who already routinely asked about CAT usage as part of their standard of care. Post‐intervention patient records were audited for the presence of the implemented CAT screening tool.

### Statistical analysis

Questionnaire responses had 5‐point Likert scale responses and were condensed into low, medium and high categories. For the analysis, the questions were grouped into the themes of knowledge, views, confidence and documentation.

Questionnaire responses and audit results were analysed utilising the Statistical Package for the Social Science (SPSS, IBM Corporation, New York, NY, USA) software. Fisher's Exact Tests and McNemar–Bowker Tests were used where appropriate and the significance threshold was set at 0.05.

Ethics approval for this study was granted by North Coast NSW Human Research Ethics Committee (LNR/14/NCC/11).

## Results

### Questionnaire results

#### Perceived knowledge

Participants improved and retained their knowledge about CATs. At post‐intervention and follow‐up stage, all participants knew the difference between a complementary and an alternative therapy. A 39% increase from baseline to post‐intervention occurred (*P *=* *0.001, McNemar Test). When compared to post‐intervention, this was not significantly different at 6 months follow‐up, suggesting improvements were sustained over time (*P *=* *1.00), see Table 2.

**Table 2:** CAT questionnaire results at baseline, post intervention and at 6 months follow up.

	Baseline *n* = 33			Post intervention *n* = 27			6 months follow‐up *n* = 21			*P* value	*P* value
	Low	Medium	High	Low	Medium	High	Low	Medium	High	Baseline versus post intervention	Post intervention versus follow up
	*n* (%)	*n* (%)	*n* (%)	*n* (%)	*n* (%)	*n* (%)	*n* (%)	*n* (%)	*n* (%)		
Knowledge											
Alternative versus Complem.	3 (9)	8 (24)	20 (61)	0 (0)	0 (0)	27 (100)	0 (0)	0 (0)	21 (100)	*P* = 0.001* ^,^ ^†^ (2‐sided)	*P* = 1.000^†^
CAT information sources	23 (70)	7 (21)	3 (9)	2 (7)	8 (30)	17 (63)	1 (5)	9 (43)	11 (52)	*P* = 0.001* df = 3	*P* = 0.453 (2‐sided)
Views											
Why CATs are used	2 (6)	3 (9)	28 (85)	1 (4)	1 (4)	25 (93)	1 (5)	1 (5)	19 (90)	*P* = 0.261 df = 3	*P* = 1.000 df = l
What CATs are used	0 (0)	0 (0)	33 (100)	0 (0)	2 (7)	25 (93)	0 (0)	2 (10)	19 (90)	*P* = 1.000	*P* = 1.000 (2‐sided)
Confidence											
CATs used	24 (73)	4 (12)	4 (12)	4 (15)	11 (40)	12 (46)	1 (5)	9 (43)	11 (52)	*P* = 0.001* df = 3	*P* = 1.000 df = 2
Advantages/disadvantages	25 (76)	4 (12)	4 (12)	6 (22)	11 (40)	10 (37)	3 (14)	7 (33)	11 (52)	*P* = 0.005* df = 3	*P* = 0.572 df = 3
Initiating discussion	21 (64)	5 (15)	7 (21)	1 (4)	13 (48)	13 (48)	3 (14)	5 (24)	13 (62)	*P* = 0.010* df = 3	*P* = 0.774 (2‐sided)
Talking about	20 (61)	6 (18)	7 (21)	2 (7)	10 (37)	15 (56)	4 (19)	3 (14)	14 (67)	*P* = 0.002* df = 3	*P* = 1.000 (2‐sided)
Documentation											
CAT in appt./tool initiate	20 (61)	4 (12)	9 (27)	2 (7)	1 (4)	24 (89)	3 (14)	6 (32)	10 (51)	*P* = 0.002* df = 3	*P* = 0.008* (2‐sided)
Screening tool to address				2 (7)	4 (15)	21 (78)	2 (10)	3 (14)	15 (75)		*P* = 0.607 df = 2
Routinely address CAT				2 (7)	1 (4)	24 (89)	2 (10)	8 (42)	9 (47)		*P* = 1.000
Record in MOSAIQ	7 (23)	3 (9)	21 (68)	2 (7)	1 (4)	23 (89)	1 (5)	0 (0)	19 (90)	*P* = 0.135 df = 2	*P* = 0.625 (2‐sided)
Effectiveness of tool	27 (87)	3 (9)	1 (3)	1 (4)	0 (0)	26 (96)	1 (5)	3 (14)	17 (81)	*P* = 0.000* (2‐sided)	*P* = 0.250 (2‐sided)
Dept. documentation	24 (77)	6 (18)	1 (3)	18 (67)	4 (15)	5 (19)	4 (19)	10 (51)	7 (33)	*P* = 0.392 df = 3	*P* = 0.058 (2‐sided)

McNemar Test unless stated otherwise

* Significant value *P* < 0.05

^†^ Fishers exact test

**Figure 2:**
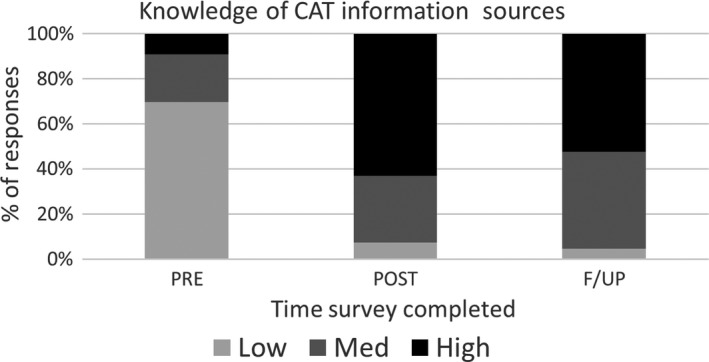
Knowledge of CAT information sources.

Significant change was measured pertaining to staff knowledge of appropriate CAT information resources. At baseline, 18 of the 27 respondents (67%) who completed the intervention had low awareness of resources available. Post‐intervention this reduced to only two participants (7%) with low and 17 (63%) with high awareness of appropriate sources (*P *=* *0.001). Only one participant remained in the low awareness category at follow‐up equating to sustained change, see Table 2 and Figure 2.

The study measured the participants' perceived knowledge about five specific CATs that patients utilise; why patients utilise it; and what the evidence‐based literature suggested. There was a significant difference (*P *<* *0.001, Fisher's exact test) in the change from baseline to post‐intervention on the five CATs measured except one aspect of high‐dose antioxidants (*P *=* *0.194), see Table 3.

**Table 3:** Participant knowledge responses on 5 commonly used CATs at baseline, post intervention and 6 months follow up.

	Baseline *n* = 33			Post *n* = 27			Follow Up *n* = 21			*P* value
	N	U	Y	N	U	Y	N	U	Y	
	*n* (%)	*n* (%)	*n* (%)	*n* (%)	*n* (%)	*n* (%)	*n* (%)	*n* (%)	*n* (%)	
**Black cohosh**										
Heard of it	15 (46)	1 (3)	17 (52)	3 (11)	0 (0)	24 (89)	0 (0)	0 (0)	21 (100)	0.000*
Patient may utilise	18 (55)	5 (15)	10 (30)	1 (4)	2 (7)	22 (81)	0 (0)	6 (29)	13 (62)	0.000*
Literature suggests	25 (76)	3 (9)	5 (15)	2 (7)	7 (26)	16 (59)	4 (19)	7 (33)	8 (38)	0.000*
**High dose anti‐oxidants**										
Heard of it	1 (3)	3 (9)	29 (88)	0 (0)	0 (0)	27 (100)	0 (0)	0 (0)	21 (100)	0.194
Patient may utilise	8 (24)	3 (9)	22 (67)	0 (0)	1 (4)	24 (89)	0 (0)	1 (5)	18 (86)	0.006
Literature suggests	13 (39)	10 (30)	10 (30)	0 (0)	5 (19)	20 (74)	1 (5)	3 (14)	15 (71)	0.000*
**Essiac**										
Heard of it	29 (88)	1 (3)	3 (9)	4 (15)	1 (4)	22 (81)	2 (10)	2 (10)	17 (81)	0.000*
Patient may utilise	30 (91)	2 (6)	1 (3)	3 (11)	7 (26)	15 (56)	3 (14)	14 (67)	2 (10)	0.000*
Literature suggests	30 (91)	3 (9)	0 (0)	4 (15)	9 (33)	12 (44)	5 (24)	11 (52)	3 (14)	0.000*
**Selenium**										
Heard of it	11 (33)	2 (6)	20 (61)	1 (4)	0 (0)	26 (96)	0 (0)	0 (0)	21 (100)	0.000*
Patient may utilise	21 (64)	4 (12)	8 (24)	2 (7)	3 (11)	20 (74)	0 (0)	7 (33)	12 (57)	0.000*
Literature suggests	24 (72)	6 (18)	3 (9)	4 (15)	4 (15)	17 (63)	2 (10)	10 (48)	7 (33)	0.000*
**Soy/lsoflavin**										
Heard of it	8 (24)	6 (18)	18 (55)	0 (0)	1 (4)	26 (96)	0 (0)	1 (5)	20 (95)	0.000*
Patient may utilise	17 (52)	8 (24)	7 (21)	0 (0)	3 (11)	22 (81)	2 (10)	7 (33)	10 (48)	0.000*
Literature suggests	23 (70)	6 (18)	3 (9)	2 (7)	6 (22)	17 (63)	4 (19)	7 (33)	8 (38)	0.000*

Fishers Exact Test

* *P* < 0.05

#### Views

Participants' views regarding the importance of patients' CAT use did not change post‐intervention. When evaluating the importance of understanding *why* patients are using CATs, 85% (28/33) fell in to the high category at baseline. A slight increase to 93% (25/27) at post‐intervention (*P *=* *0.261) and 90% (19/21) at follow‐up (*P *=* *1.000) occurred. Most participants' thought that knowledge of *what* CATs patients are using was of high importance when measured at baseline (100%), post‐intervention (93%) and at follow‐up (90%), (*P* = 1.000, McNemar Test), see Table 2.

Most participants indicated that radiation oncologists should address patients' CAT use, scoring 96–97% across all three time points (*P *=* *1.000, Fishers Exact Test), see Table 4.

**Table 4:** Participants' views on which health professionals should address patients CAT use.

	Pre *n* = 33	Post *n* = 27	Followup *n* = 21	*P* value
Health professional group	*n* (%)	*n* (%)	*n* (%)	Fishers exact
Allied health	23 (70)	23 (85)	16 (73)	0.377
Nurse	26(79)	23 (85)	18 (82)	0.934
Radiation Oncologist/Registrar	32 (97)	26(96)	21 (96)	1
Radiation therapist	20(61)	20(74)	16 (73)	0.524

* *P *< 0.05.

#### Confidence

Participants' confidence in their understanding of patients' CAT use increased post‐intervention. Post‐intervention 23 of 27 participants were in the mid‐high category of confidence; this was statistically significant (*P *=* *0.001). Nineteen participants who completed the intervention had low confidence at baseline and after attending the intervention education session only four remained in the low confidence category. Participants post‐intervention and follow‐up responses were also compared. There was no change indicating that levels of confidence gained in understanding what CATs patient utilised retained over the 6 months (*P *=* *1.00), see Table 2.

Eight respondents from 33 (24%), rated themselves as mid‐high confidence in understanding the advantages and disadvantages of CATs patients utilise at baseline. This increased significantly post‐intervention to 78% where 21 of 27 felt they were in mid‐high categories (*P *=* *0.005) and this change was sustained (*P* = 0.572), see Table 2.

Confidence in initiating discussion with patients about their CAT use improved from baseline through to follow‐up. Sixteen participants who completed the intervention responded in the low category with little confidence initiating discussion and only one remained in this category. The remainder of participants were spread equally with 13 in each of the mid and high categories 96% (26/27) (*P *=* *0.001). The follow‐up indicated no significant change (*P *=* *0.774) hence confidence in initiating discussion was sustained, see Table 2.

**Figure 3:**
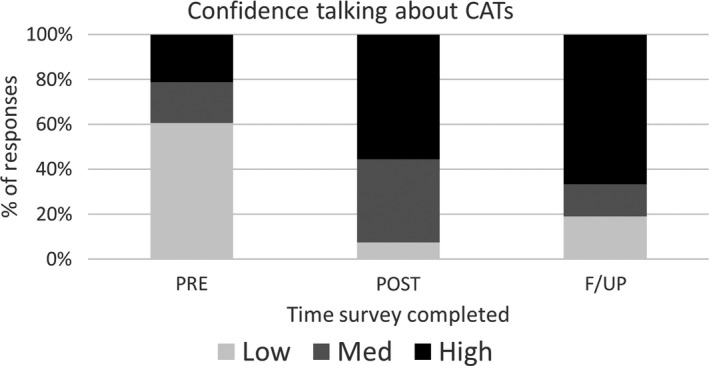
Confidence talking about CATs.

Initially 15 participants who completed the intervention were in the low category of confidence talking to patients about their CAT use. Only two remained in this category post‐intervention and 93% of participants were of mid to high confidence (*P *=* *0.002). This change was sustained at the follow‐up (*P *=* *1.000), see Table 2 and Figure 3.

#### Documentation

The participants' responses revealed no significant change regarding documenting CATs in the patient record. Responses were high across all three time points (68%, 89%, 90%, *P *=* *0.153). The CAT screening tool was perceived to be effective at intervention (78%) and at follow‐up (75%, *P *=* *0.607).

There was little change pre‐ and post‐intervention with participants' satisfaction of the way the centre currently documents CAT use. At baseline 24 responses indicated low levels of satisfaction and at post intervention there were 18 similar responses (P=0.392). Six months later there was no significant change (*P*=0.058), see Table 2.

### Audit results

Patient records were audited pre‐intervention (108/196) and post‐intervention (88/196), 19% (38/196) were head and neck patients. Post‐intervention, 50% (44/88) of patient records had the screening tool present, see Table 5.

**Table 5:** Patient record CAT use audit results with consideration to head and neck (H&N) patients.

	Pre intervention *n* = 108						Post intervention *n* = 88					
	% of *n*		% of *n*				% of *n*		% of *n*			
Mentions	Non H&N	(non H&N)	H&N	(H&N)	Combined % of n		Non H&N	(non H&N)	H&N	(H&N)	Combined % of n	
0	75	92	18	69	93	86	46	61	7	58	53	60
1	3	4	4	15	7	7	22	29	5	42	27	31
2	4	5	4	15	8	7	4	5	0	0	4	5
3							2	3	0	0	2	2
4							1	1	0	0	1	1
5							1	1	0	0	1	1
**Total**	**82**		**26**		**108**		**76**		**12**		**88**	

#### CAT mentions in the patient record

Prior to the intervention, 86% of patients (93/108) had no mention of CATs in their patient notes. Post‐intervention, the number of combined patients with no mentions dropped to 60% (53/88). This was more in non‐head and neck patients (92%, 75/82) and less in head and neck patients (69%, 18/26). In non‐head and neck, the patient records documentation increased by 31% (from 9% to 40%) and in head and neck patients 11% (from 31% to 42%), see Table 5.

The change in documentation was significant overall (*P *=* *0.001, Fisher's Exact test). Analyses also found a small increase in the number of CAT mentions per patient record, from 2 to 5, see Table 5.

## Discussion

The aim of this study was to improve the radiation therapy staff knowledge, documentation, confidence and views of cancer patients' use of CATs. The need for increasing staff awareness, knowledge and discussion of CAT use with cancer patients has been previously established in the literature. This study demonstrated that the same issues were present in the Lismore radiation therapy centre. A gap in the literature was addressed and this study provided a solution rather than repeating previously published studies. There is little evidence or guidance for radiation oncology treatment centres to improve these aspects. This research was designed to address the issues locally and rather than repeating previously published literature, to build on previous work and provide a practical solution.

### Views

The need for health professionals to develop balanced attitudes and knowledge is paramount due to the prevalence of CAT use by patients.^8^ Staff responses were consistently high when asked if they felt it was important to understand what CATs are used by patients and why. This demonstrated that the radiation oncology staff already understood the importance of patients' CAT use. From this base, the next step was to normalise CAT discussion and documentation within the centre.

### Education, knowledge and providing resources

There is a need for staff to have a greater awareness of patients' CAT use and clinicians want access to evidence‐based information and increased communication with patients.^9^, ^12^ Knowledge of CATs is part of being a well‐rounded practitioner, yet they are not likely to know the answers to patients' questions without education.^8^ The literature shows that patients understood health professionals may lack the necessary knowledge, but feel they are the best source of advice surrounding their own CAT use.^4^ Patients will continue to seek advice from radiation therapy staff therefore equipping staff with knowledge can only help to increase the quality of discussion with patients about CAT use. When staff were provided with education and information, the study found that there was an increase in most aspects of perceived knowledge. Study participants did not initially know some CATs that patients used or the reasons for taking them. High‐dose antioxidants were already known to participants but the reason a patient may utilise them and what the literature suggested increased after the education^4^, ^21^, ^22^, ^23^. The education session not only introduced new CATs but more importantly increased knowledge of previously known CATs. This indicates that the education session would be of benefit to new staff upon commencement of employment regardless of experience.

Cancer patients obtain information from varied sources; however, literature emphasises the importance of providing patients with evidence‐based CAT information.^7^, ^9^, ^15^ The Clinical Oncological Society of Australia has requested oncology staff become familiar with reputable evidence‐based resources for themselves and patients.^3^ Oh et  al, suggested that assistance is required to provide staff with updated evidence‐based information.^7^ This study demonstrated an effective solution to providing radiation therapy staff with evidence‐based resources and the confidence to use them.

### Disclosure, discussion and documentation

It is well‐established that clinicians are strongly encouraged to routinely ask patients about CAT usage due to the potential risks; however, a lot of patients do not disclose their CAT use.^3^, ^6^, ^9^, ^12^ Furthermore, a 2007 study of Australian radiation therapy centres stated only 44% obtain details of CAT use.^15^ Increased staff confidence helps build rapport and open communication between patients and practitioners. Enhancing this relationship facilitates the ability to gain in‐depth information and ultimately advise patients better. Patients have expressed they would prefer to discuss their use of CATs with their oncologist and valued when their CAT use was taken as part of their history. Due to the lack of evidence‐based research, there are no definitive answers to some questions patients may ask about their CAT use. Health professionals, however, should encourage open communication and discuss CAT use with patients. This allows for informed consent whereby the information is provided by current evidence. Patients also understood that oncologists did not have time to discuss CAT use in depth.^17^ Implementation of the CAT screening tool gave radiation oncologists the opportunity to discuss CAT use with their patients. The screening tool could be used as a prompt to initiate discussion as well as providing the oncologist with a list of CATs the patient was using. Initial consult is an ideal time for CAT use to be identified and discussed rather than during treatment. The limited time available at consult could be spent on those CATs that require discussion due to potential harm to the patient. Complementary and alternative therapies may be endorsed more often by nurses and radiation therapists than radiation oncologists in radiation oncology treatment centres.^15^ Therefore, all staff required access to the screening tool, not just radiation oncologists. Electronically filing the screening tool in each patient's record allowed access by all staff at any point throughout the patients' journey including the initial radiation oncologist consult.^17^ Optimal use of the screening tool allows staff to both initiate conversation with patients and to focus discussion on those CATs that require attention to reduce indirect harm to patients or possible treatment interactions.^9^, ^18^ This study provided a solution to aid CAT discussion and open communication between staff and patients which is paramount.^3^, ^5^, ^7^

Introducing a CAT screening tool in conjunction with an education session has noticeably increased the documentation of CAT use in the centre. Most participants' felt they already documented effectively before the intervention; however, the patient record audit demonstrated that there was a significant improvement in CAT mentions in the patient record. This study indicated a better rate of documentation than previous published results and falls in line with, or higher in some cases, than other rates of disclosure and documentation published.^10^, ^12^, ^13^

### Future opportunities

There is potential to transfer and apply the evidence gathered from this study to other radiation therapy or cancer centres. Opportunities exist for other radiation therapy centres to increase their disclosure and documentation rates by applying a similar strategy. Opportunity awaits further radiation therapy researchers to leverage off this study and the idea of standardised CAT screening could be explored. There is scope for future CAT clinical trials to build on the current limited body of knowledge.

## Limitations

The small sample did not allow for participants to be split into separate groups according to discipline, and therefore take into account different roles, knowledge and practices. This study detected a significant change with a small sample size that demonstrates the strength of the results.

Staff departures and the inability to attend the education intervention excluded some participants from continuing in the study; however, the response rate was extremely high.

There was no existing validated tool; therefore the questionnaire was developed by the principal investigator. A risk with a custom designed questionnaire is the potential to coerce participants' responses. The questionnaire was tested by a non‐participant to mitigate this and avoid bias.

## Conclusion

This study has demonstrated that staff education and the implementation of a screening tool increased and sustained staff knowledge and confidence discussing CAT use with patients and CAT documentation, in a regional radiation oncology treatment centre. This study provided a solution to initiate CAT discussion, open communication between staff and patients and the documentation of patients CAT use. This study changed the way CAT use is managed in the Lismore radiation oncology treatment centre in order to assist patients more effectively. Patients were given the opportunity to talk about their CAT use with staff who had improved knowledge and confidence. The need to provide education to increase and encourage open communication between clinicians and patients was addressed and this is the first published study to provide a practical and validated solution to this issue. It provided a solution to a gap in the published literature providing education and resources to radiation oncology staff.

## Supporting information


**Data S1** **.** Complementary and alternative therapy (CAT) use questionnaire at North Coast Cancer Institute Lismore, Radiation Oncology Unit.
