# Supplementary material for: Complementary and alternative therapy use in a regional radiation oncology treatment centre: Can staff knowledge, views, confidence and documentation be improved?
Source: J Med Radiat Sci. 2019 Aug 8;66(3):191–9. doi: 10.1002/jmrs.344 (PMC6745355; doi:10.1002/jmrs.344)
Supplement: Supplementary file 1 — Data S1 . Complementary and alternative therapy (CAT) use questionnaire at North Coast Cancer Institute Lismore, Radiation Oncology Unit. [file JMRS-66-191-s001.docx]

**
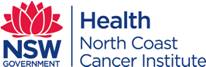
**
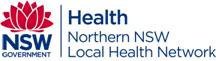


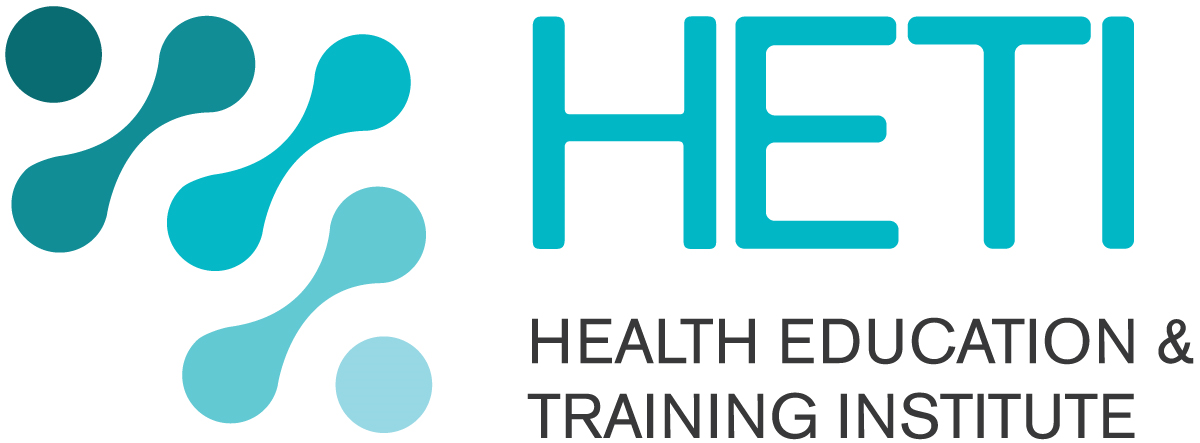


**Complementary and Alternative Therapy (CAT) Use @ North Coast Cancer Institute Lismore, Radiation Oncology Unit.**

Post Intervention Questionnaire

NAME:____________________

*Please circle one answer per question*

1. Discipline in the department

| Radiation Therapist | Nurse | Radiation Oncologist/ RO Registrar | Allied Health ( Dietician, Social Worker, Speech Pathologist) |
| --- | --- | --- | --- |

1. Do you know the difference between a complementary and an alternative therapy?

| Yes | Unsure | No |
| --- | --- | --- |

1. Do you think it’s important to understand why patients are using these CATS?

| Very Important | Important | Moderately Important | Of Little Importance | Unimportant |
| --- | --- | --- | --- | --- |

1. Do you think it’s important to understand what CATS patients are using?

| Very Important | Important | Moderately Important | Of Little Importance | Unimportant |
| --- | --- | --- | --- | --- |

1. How confident are you in your understanding of what CATS your patients utilize?

| Very Confident | Somewhat Confident | Confident | Barely Confident | Not Confident |
| --- | --- | --- | --- | --- |

1. How confident are you in your understanding of what the advantages/disadvantages of such CATS your patients utilize?

| Very Confident | Somewhat Confident | Confident | Barely Confident | Not Confident |
| --- | --- | --- | --- | --- |

1. The table below provides a list of commonly used CATS provided by a literature search.

Please circle Y (Yes), U (unsure) or N (No) in each of the columns for each CAT

|  | Have You Heard of this CAT? | Do you know why a patient may utilise it? | Do you know what the literature suggests? |
| --- | --- | --- | --- |
| Black Cohosh  (eg Remifemin) | Y U N | Y U N | Y U N |
| High dose Antioxidants | Y U N | Y U N | Y U N |
| Essiac | Y U N | Y U N | Y U N |
| Selenium | Y U N | Y U N | Y U N |
| Soy/isoflavin | Y U N | Y U N | Y U N |

In your experience what is a commonly ingested CAT used by patients not already

Mentioned on this list?____________________________________

1. Are you aware of appropriate information sources available to help you when discussing CAT use with patients?

| To a Great Extent | Above Adequate | Somewhat | Very Little | Not at All |
| --- | --- | --- | --- | --- |

1. How confident are you in initiating discussion about CAT use with patients?

| Very Confident | Somewhat Confident | Confident | Barely Confident | Not Confident |
| --- | --- | --- | --- | --- |

1. How confident are you in talking with patients about their CAT use?

| Very Confident | Somewhat Confident | Confident | Barely Confident | Not Confident |
| --- | --- | --- | --- | --- |

1. How effective will the screening tool be in initiating discussion with patients about their CAT use?

| Extremely Effective | Very Effective | Adequate | Possibly Effective | Not Effective |
| --- | --- | --- | --- | --- |

1. Do you think the screening tool will help the way your department addresses CAT use with your patients?

| Definitely | Very Probable | Probably | Possibly | Probably Not |
| --- | --- | --- | --- | --- |

1. Will the implementation of the screening tool and the update to the Electronic Medical Record (MOSAIQ) ensure that CAT use will be routinely addressed for all patients?

| Definitely | Very Probable | Probably | Possibly | Probably Not |
| --- | --- | --- | --- | --- |

1. Who should discuss CAT use with patients? (Circle multiple answers if necessary)

| Radiation Therapist | Nurse | Radiation Oncologist/ RO Registrar | Allied Health ( Dietician, Social Worker, Speech Pathologist) | Other |
| --- | --- | --- | --- | --- |

1. When patients discuss their CAT use do you record it in their Electronic Medical Record (MOSAIQ)?

| Always | Usually | About Half the Time | Seldom | Never |
| --- | --- | --- | --- | --- |

1. Do you think the screening tool will be an effective way of documenting CAT use in the department?

| Definitely | Very Probable | Probably | Possibly | Probably Not |
| --- | --- | --- | --- | --- |

1. Are you satisfied with the way your department currently documents CAT use amongst patients?

| Extremely satisfied | Very satisfied | Satisfied | Somewhat satisfied | Not satisfied |
| --- | --- | --- | --- | --- |
